# Marginal adaptation analysis of CAD/CAM resin crown with non-invasive methods

**DOI:** 10.1007/s00784-025-06215-6

**Published:** 2025-02-26

**Authors:** Chen Zeng, Tomoko Tabata, Rena Takahashi, Masaomi Ikeda, Junichi Shinagawa, Hisaichi Nakagawa, Yumi Tsuchida, Shunsuke Takano, Yasunori Sumi, Yasushi Shimada

**Affiliations:** 1 Department of Cariology and Operative Dentistry, Graduate School of Medical and Dental Sciences, Institute of Science Tokyo, 1−5−45 Yushima, Bunkyo−ku, Tokyo, 113−8549 Japan; 2 Department of Oral Biomedical Engineering, Graduate School of Medical and Dental Sciences, Institute of Science Tokyo, 1−5−45 Yushima, Bunkyo−ku, Tokyo, 113−8549 Japan; 3 Department of Digital Dentistry, Graduate School of Medical and Dental Sciences, Institute of Science Tokyo, 1−5−45 Yushima, Bunkyo−ku, Tokyo, 113−8549 Japan

**Keywords:** SS-OCT, Silicone replica, Marginal adaptation, CAD/CAM

## Abstract

**Objectives:**

This study compared the silicone replica method with swept-source optical coherence tomography (SS-OCT) to analyze marginal adaptation and investigated the effect of the light incidence angle of SS-OCT on measurement precision.

**Materials and methods:**

A typodont-prepared mandibular right first molar was scanned using an intraoral scanner (Trios 3). Fourteen crowns were fabricated from CAD/CAM resin blocks (Katana Avencia P) using a 5-axis milling machine (DWX-50). Marginal adaptation at the buccal, lingual, mesial, and distal points was assessed using the silicone replica method and SS-OCT at light incidence angles of 60°, 75°, and 90°. Statistical comparisons were performed using two-way analysis of variance (ANOVA) and t-tests with Bonferroni correction, and t-tests at a significance level of 0.05.

**Results:**

At 60°, SS-OCT showed significantly larger marginal discrepancies than the silicone replica method at the buccal, lingual, and mesial points (*p* < 0.05). At 75°, only the lingual point showed a significantly larger value than the silicone replica method (*p* < 0.05). At 90°, no significant differences were observed between the SS-OCT and silicone replica methods (*p* > 0.05). Marginal discrepancy values increased as the angle changed from 90° to 75° to 60°, with significant differences between 60° and 75° and between 60° and 90° at the buccal and lingual points (*p* < 0.05).

**Conclusions:**

SS-OCT is a viable alternative to the silicone replica method for assessing marginal adaptation at an incidence angle of 90 °.

**Clinical relevance:**

SS-OCT, a non-invasive method, has the potential to be applied clinically for evaluating marginal fit in indirect restorations in vivo.

## Introduction

In the rapidly advancing field of modern dentistry, CAD/CAM technology has significantly enhanced aesthetic restorations [1]. This innovative technique not only meets patients’ high aesthetic expectations but also offers superior light transmission, closely mimicking the color and translucency of natural teeth [2–6]. The success of these restorations depends critically on their marginal adaptations. Poor adaptation can lead to a cascade of issues, including cement deterioration, microleakage, tissue inflammation, recurrent decay, and ultimately, restoration failure [7, 8].

Prior research has outlined several approaches for measuring marginal adaptation, including the direct-view technique, cross-sectioning technique, replica method, profile projection, digital impression, and microcomputed tomography [9–15]. The silicone replica method is one of the techniques most commonly used for this purpose. The replica method produces a silicone duplicate of the space between the dental prosthesis and the prepared tooth [16]. The replica was then dissected and examined under a microscope to evaluate the prosthesis alignment and precision.

Optical coherence tomography (OCT) is a noninvasive imaging method that utilizes light reflection to generate high-resolution cross-sectional images of internal structures [10, 17, 18]. Swept-source OCT (SS-OCT), an advanced version, employs a wavelength-tuned laser source and offers enhanced resolution and faster scanning [19]. OCT operates based on low-coherence interferometry principles and provides detailed depth information [20]. This technique has been applied in various dental diagnostics including caries and periodontal tissues [21, 22]. In addition, literature indicates that OCT can be used to assess composite restorations, adhesives and indirect restorations [23–30]. The accuracy of the OCT images can vary depending on the angle of the sample relative to the light source, as the vertical length changes with different cross-sectional angles [31]. Therefore, it is crucial to investigate the precision of the OCT images when the samples are positioned at various angles relative to the OCT laser light source.

Numerous factors affect the marginal adaptation of CAD/CAM restorations, including margin design, die spacer thickness, cement type, and cementation method employed [13, 15, 32–37]. In this study, marginal adaptation was defined as the distance between the outermost points of the abutment and the crown. McLean et al. [37] reported a maximum clinically acceptable marginal gap of 120 𝓊m, while Boening et al. [38] described a clinically acceptable marginal gap of 100 𝓊m to 200 𝓊m. Both the distance between the OCT’s near-infrared (NIR) light source and the incident angle of the light upon the specimen are statistically significantly related to the OCT images [39]. Increasing the angle of incidence lengthens the sample’s light propagation path, reducing imaging resolution. Geometric distortion occurs when the incidence angle changes the image’s transverse-to-longitudinal ratio [40].

This study aimed to compare the silicone replica method with the SS-OCT method for analyzing marginal adaptation and to investigate the impact of the SS-OCT light incidence angle on this analysis. The null hypothesis was that the SS-OCT method would not differ from the silicone replica method in measuring marginal adaptation and that the angle of light incidence in SS-OCT would not affect the analysis of marginal adaptation in CAD/CAM-fabricated resin crowns.

## Materials and methods

**Fig. 1:**
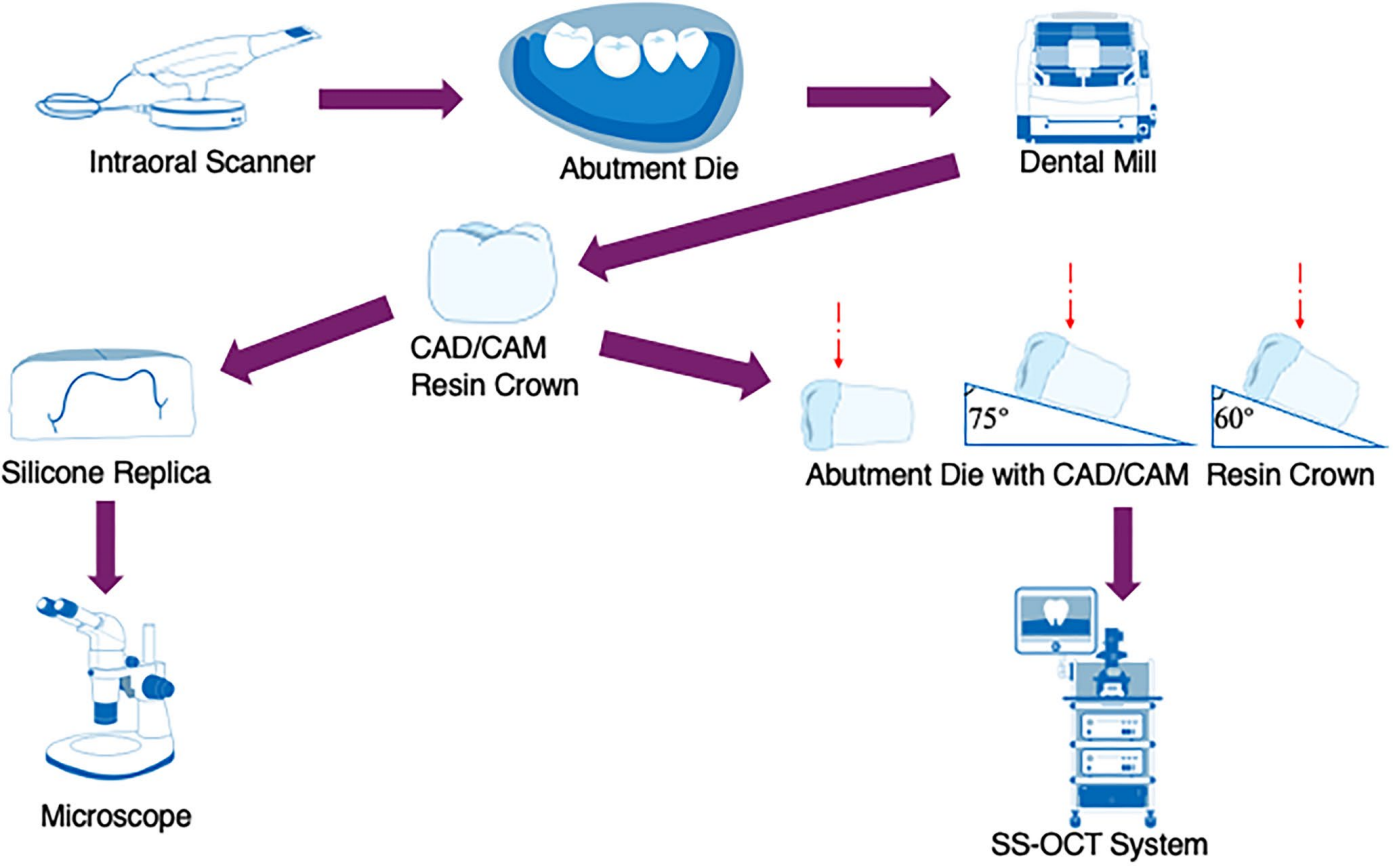
Schematic illustration for this study. Red dotted lines indicate the incidence of the near-infrared light

Schematic illustration for this study is presented in Fig. 1.

### Specimen preparation

A plastic mandibular right first molar die for a jacket crown (A55A-461, Nissin, Kyoto, Japan) was mounted on a typodont standard model (500 H-1 M, Nissin, Kyoto, Japan) and scanned using an intraoral scanner (Trios 3, 3Shape, Copenhagen, Denmark). The crown shape was then designed using CAD software (S-WAVE Dental System, Shofu, Kyoto, Japan) by selecting an appropriate library template, with the cement space set at 150 μm for the occlusal surface and axial wall, and 50 μm for the margin. Fourteen crowns were fabricated using CAD/CAM resin blocks (Katana Avencia P Block, Kuraray Noritake Dental, Tokyo, Japan) and milled using a 5-axis milling machine (Dental Mill, DWX-50, Roland, Osaka, Japan) according to the manufacturer’s specifications.

### Measurement of marginal adaptation using silicone replica method

**Fig. 2:**
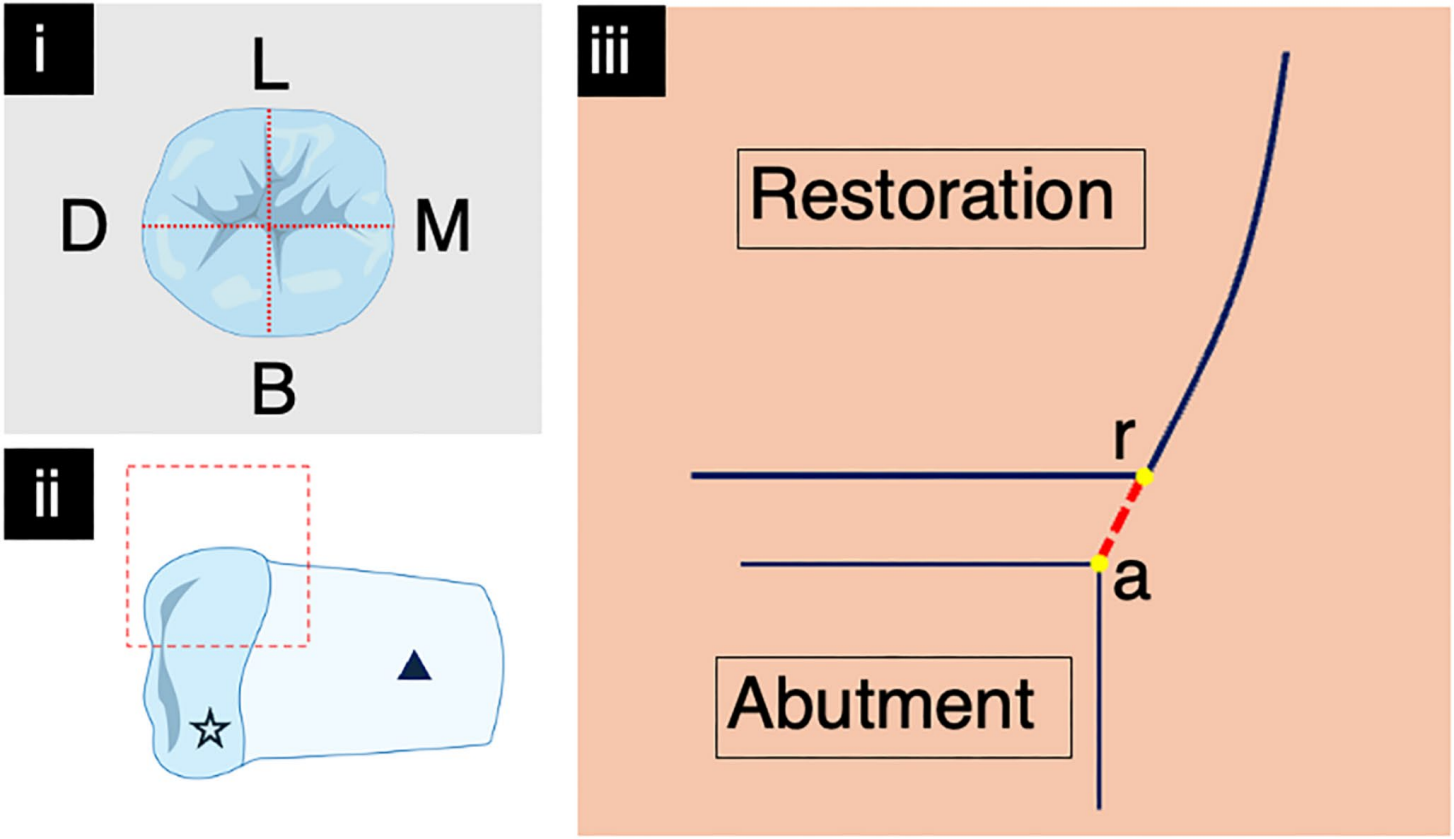
(**i**) The dotted lines indicate the directions of four cross-sections from the occlusal view. B: buccal; D: distal; L: lingual; M: mesial. (**ii**) The dashed square indicates the range of a single scan of SS-OCT. The pentagram symbol indicates the restoration, and the triangular symbol indicates the abutment die. (**iii**) Marginal adaptation. The marginal discrepancy is defined as the distance from the outmost point of the restoration (r) to the outmost point of the abutment (a)

Each crown was filled with light-body occlusal contact and fit-checking material (GC Blue Silicone, GC, Tokyo, Japan) and then placed onto the abutment die. A 2 kg weight was applied from the occlusal direction for 5 min. Once the light-body silicone was set, the resin crown was removed, leaving a thin film of light-body silicone on the die. Heavy-body hydrophilic vinyl polysiloxane (Exafine Regular Hard Type, GC, Tokyo, Japan) was then applied over the light-body silicone film as an outer layer. After the heavy-body vinyl polysiloxane had set, the two-layer replica was removed from the die, and the heavy-body vinyl polysiloxane was placed into the intaglio surface of the two-layer replica until it set. The three-layered silicone replica was segmented buccolingually and mesiodistally by using a razor blade (FAS-10; Feather, Osaka, Japan). Each segment of the silicone replica was photographed using an optical microscope (SMZ1000, Nikon, Tokyo, Japan) at 28x magnification to obtain cross-sectional images of the buccal, lingual, mesial, and distal points (Fig. 2). The buccolingual and mesiodistal cutting lines were passed through the midpoint of the die’s occlusal surface and were perpendicular to each other, bisecting the buccal, mesial, distal, and lingual sides.

### SS-OCT Observation

The SS-OCT system (IVS-2000, Santec, Aichi, Japan) utilized a central wavelength ranging from 1,260 nm to 1,360 nm, centered at 1,310 nm, and generated NIR light at a sweep rate of 20 kHz. In the air medium, the device achieved a depth resolution of less than 7 μm, while the axial and lateral resolutions were 11 μm and 17 μm, respectively. The probe connected to the interferometer had a power of 5mW, which was within the safety limit of the American Standard Institute. The laser source emitted from the probe was directed onto the sample at the desired location in the X and Z dimensions. The backscattered light carrying information from each single scan point of the sample was returned to the system, digitized on a time scale, and analyzed in the Fourier domain to disclose the depth information (A-scan) of the sample. The A-line and 2D frame A-scan rates are 20,000 lines/second and 260 lines/frame. By combining a series of A-scans in a linear fashion across the sample, a cross-section (B-scan) was obtained. Finally, cross-sectional images could be created by converting the B-scan raw data into a greyscale image with 2001 × 1019 pixels [41–43]. The crown and abutment dies were observed in an uncemented state. The crown was connected to the abutment die using a small amount of clear vinyl polysiloxane (Exaclear, GC), ensuring that the material did not encroach the observation area while still providing stability to the crown and abutment die. Wedge-shaped supports with inclinations of 15° and 30° were used to vary the light incidence angle. The handheld scanning probe fixed on the OCT device was 5 cm away from the specimen and aligned perpendicularly, and the probe could only be moved in the vertical direction during scanning [44]. Subsequently, all samples were observed using the SS-OCT system with a scanning range of 8 × 8 mm from the buccal, lingual, mesial, and distal sides at three different light incidence angles: 60°, 75°, and 90° (Fig. 2), at an intensity of − 10 dB and an A-scan average of one. A tailored pedestal was employed to support the abutment die and to denote the orientation for the different cross sections, short lines were drawn with a marker pen in all four directions of the abutment die in accordance with the orientation indicated on the pedestal. Each sample was moved in a mid-range direction across the laser beam until it aligned with the short line marker on the abutment die, at which point the clearest cross-sectional B-scan image was selected and captured from a series of real-time B-scan previews.

### Image processing

**Fig. 3:**
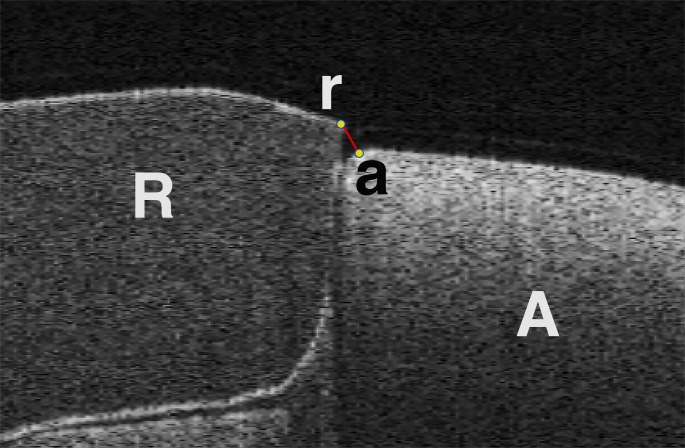
Exemplary of 2D SS-OCT image. The marginal discrepancy is defined as the distance from the outmost point of the restoration (r) to the outmost point of the abutment die(a). R: restoration, A: abutment die

All two-dimensional (2D) SS-OCT images were saved in CSV format at a pixel size of 16,000 × 7,481 μm. ImageJ (version 13.0.6, National Institutes of Health, Bethesda, MD, USA) was used to analyze all microscopic and 2D SS-OCT images. Marginal discrepancy was defined as the distance between the outermost points of the abutment and restoration [45] (Fig. 3).

### Statistical analysis

The number of CAD/CAM resin crown (*n* = 14) were determined by data of the pilot study with statistical software (G*power 3.1.9.2) and the α error and statistical power (1-β error) were set to 0.05 and 0.8, respectively. After the normality and variance of the SS-OCT data were confirmed using the Shapiro-Wilk test and Levene’s test, respectively, two-way ANOVA and t-tests with Bonferroni correction were applied. Subsequently, comparisons between the replica method and SS-OCT at each degree and position were conducted using t-tests. Statistical analyses were conducted at a significance level of 0.05, using statistical software (SPSS ver. 27.0 for Windows, IBM, Chicago, IL, USA).

## Results

**Fig. 4:**
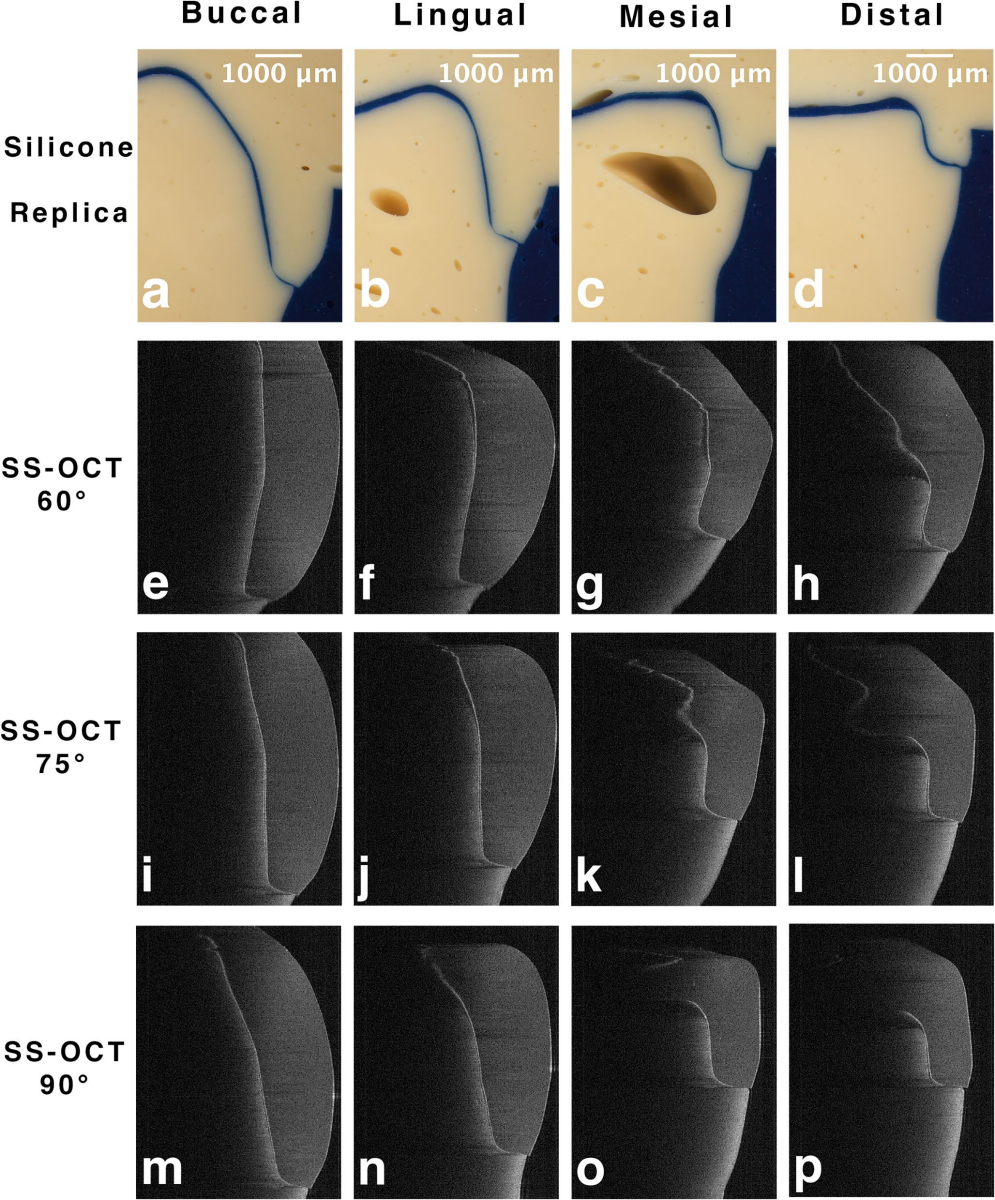
Microscopic images and 2D SS-OCT images of buccal, lingual, mesial, and distal points. **a**-**d**: microscopic images of silicone replica. **e**-**h**: 2D SS-OCT images of 60° NIR light incidence. **i**-**l**: 2D SS-OCT images of 75° NIR light incidence. **m**-**n**: 2D SS-OCT images of 90° NIR light incidence

Figure 4 shows the buccal, lingual, mesial, and distal images of the silicone replica and SS-OCT methods with 60°, 75°, and 90° light incidences. Table 1 shows the results of the marginal discrepancy between the silicone replica and SS-OCT methods with 60°, 75°, and 90° light incidence. On 60° SS-OCT, the marginal discrepancy values were significantly larger than those on the silicone replica method at the buccal, lingual, and mesial points (*p* < 0.05). On 75° SS-OCT, only the lingual point showed significantly larger values than the silicone replica method (*p* < 0.05); the other three points showed no differences (*p* > 0.05). For 90° SS-OCT, there were no statistically significant differences between the silicone replica and SS-OCT methods at any point (*p* > 0.05).

**Table 1:** Marginal discrepancy of silicone replica method and SS-OCT method with 60°, 75°, and 90° light incidences in µm

	Buccal	Lingual	Mesial	Distal
Silicone Replica	142 ± 7.71*	134± 6.84*^,^ **	146 ± 4.50*	134 ± 8.67
60° SS-OCT	211± 12.1^a,^*	220 ± 8.93^a,^ *	164 ± 7.39^b, C,^ *	156 ± 11.0^b, D^
75° SS-OCT	141 ± 9.12^c, A^	173 ± 9.59^c, B,^ **	153 ± 6.27^c, C^	141 ± 7.99^c, D^
90° SS-OCT	139 ± 8.13^d, A^	157 ± 10.8^d, B^	142 ± 8.30^d, C^	139 ± 8.84^d, D^

All values are the mean ± SEM. Within the same row of SS-OCT at each light incidence, the same lowercase letters indicate no significant difference (*p* > 0.05). Within the same column, the same uppercase letters indicate no significant differences in SS-OCT (*p* > 0.05). “*” and “**” show significant differences between the replica methods and SS-OCT at each degree and position (*p* < 0.05)

At the buccal, lingual, mesial, and distal points, the SS-OCT values tended to increase as the angle changed from 90° to 75° to 60°. At the buccal and lingual points, there were significant differences in the marginal discrepancy data between 60° and 75°, and between 60° and 90° on SS-OCT (*p* < 0.05). When comparing the four points (buccal, lingual, mesial, and distal) at 60°, 75°, and 90° of SS-OCT, there were no significant differences among the four points at 75° and 90° (*p* > 0.05). At 60° SS-OCT, while there were no significant differences between buccal and lingual and between mesial and distal (*p* > 0.05), significant differences were found in all other combinations (*p* < 0.05).

## Discussion

The purpose of this experiment was to evaluate the performance of OCT in measuring the marginal adaptation of indirect restorations. The film thickness and marginal adaptation significantly affect the longevity and durability of indirect restorations [38, 46]. Inadequate marginal fit and gaps in the crown leads to cement dissolution, microbial colonization, deposition of dental biofilms, gingival inflammation, recurrent caries, increased periodontal pockets, and bone loss [8]. Kokubo et al. [46] emphasize that clinicians must be aware that the position of the crown and the type of restoration will affect a considerable range of marginal fit. A proper marginal fit ensures long-term results of an indirect restoration’s length of service and persistence. In clinical situations, it is difficult to directly determine the film thickness of indirect restorations.

OCT can noninvasively create cross-sectional images of biomaterials and organs using light. This non-ionizing imaging method has shown significant potential in dentistry, with applications ranging from the mineral content estimation of hard-tooth structures to incomplete crown cracks [17, 47–50]. The backscattered light intensity was recorded based on the depth (axial position) of the tissue. Low-coherence interferometry is employed to capture the intensity of backscattered light, providing positional information with a resolution of up to 20 microns [51]. Each substance, whether solid, liquid, or gas, has a refractive index. Successful imaging relies on the incident light reflected by SS-OCT between two media with different refractive indices [43, 51]. When the refractive index of an object is greater than one, it appears enlarged in the direction of the NIR light incidence in an SS-OCT cross-sectional image [47]. However, when investigating the marginal adaptation, the measurement points were located at the edges of the abutment tooth and restoration. Consequently, the refractive index had a smaller impact on these specific points.

In SS-OCT observations, the scanning angle relative to the sample influences the length and width of the resulting image [17]. In clinical situations, when the anatomical shape of the tooth prevents the perpendicular NIR light, an image is captured at an oblique angle. When using SS-OCT with an incident angle of < 90°, the NIR light must travel a longer path to pass through the edge of the resin crown. This increased distance also makes it more challenging to obtain a clear image because the edge of the resin crown is farther from the light source than at a 90° angle.

The anatomical shape of the tooth and length of the restoration contour also affected the SS-OCT image. The abutment die used in the experiment was a right mandibular first molar, accompanied by 14 CAD/CAM fabricated resin crowns; each sample was scanned from four directions. The resin crowns were similar in shape to natural teeth, with the mesial and distal sides having a shorter and straighter occlusal-cervical profile than the buccal and lingual sides (Fig. 4). The angle between the incident light and surface of the object affects the direction of light refraction. The distance that the NIR light must travel is greater when passing through the edge of a restoration at an obtuse angle than when passing through the edge of a restoration at an acute angle. This implies that the distance between the outermost point of the restoration and the outermost point of the abutment increases [31]. As a result, the buccal and lingual sides on 60° SS-OCT showed larger values than those obtained using the silicone replica method and 75° and 90° SS-OCT in this experiment. However, there was no statistically significant difference between the results of the SS-OCT and silicone replica methods when the incident light was perpendicular to the sample surface. This finding underscores the distinctive efficacy of the 90° incidence of the SS-OCT method, as it yielded outcomes that surpassed those of the other SS-OCT groups and attained an accuracy level comparable to that of the silicone replica method (Table 1). The null hypothesis was partially rejected based on the points discussed above.

The mean values of marginal adaptation in this study were within the acceptable range, except for the buccal and lingual values at a 60° angle of incidence in SS-OCT observation. The angle of incidence of the NIR light significantly affected the measurements. In the clinical setting, determining the tooth axis and probe tilt is crucial for accurate marginal adaptation assessment using OCT. As noted by Kikuchi et al. [31], it would be desirable to have software that can correct the measured length based on the angle of incidence of NIR light, which can be used to improve the measurement accuracy, particularly for teeth with large tilt angles. This feature should be considered in clinical OCT systems. In this in vitro experiment, the SS-OCT probe was temporarily immobilized above a horizontal sample stage that could be moved up and down, left and right, and forward and backward. The specimen loaded in the pedestal is placed on the sample stage for observation. The customized pedestal of the abutment die ensures that the NIR light is perpendicular to the specimen’s surface, and the short line marker ensures no translation or rotation of the specimen while observing the four target cross-sections. In addition, the method of using feature point matching for image correction in oral digital impressions has potential to help correct OCT data affected by incidence angle differences.

Although the silicone replica method is widely used, it has several limitations in assessing marginal adaptation [16, 52]. It relies on two-dimensional (2D) analysis with limited measurement points, typically only two to four per sample [14]. Thin silicone films may deform or tear during removal, affecting the measurement accuracy [38]. Moreover, the silicone material type and measurement process can affect the method’s accuracy [53].

Currently, there are no absolute methods for measuring marginal discrepancies in restorations. The lack of standardization makes inter-study comparisons difficult [9, 10, 35]. In this study, the results of the marginal discrepancy measurements were consistent between the replica and SS-OCT methods at 90° incidence, both of which are recognized as noninvasive techniques. These methods are promising and effective tools for detecting marginal adaptation between crowns and abutment teeth.

## Conclusion

Based on the results of this experiment, the following conclusions can be drawn:

The SS-OCT method can serve as a viable alternative to the silicone replica method for assessing marginal adaptation when the observation incidence is set at 90° towards the CAD/CAM resin crown. However, it is important to note that the angle of near-infrared light incidence significantly affects the precision of the marginal adaptation analysis. Furthermore, the marginal discrepancy in the CAD/CAM resin crowns demonstrated good consistency across the four observed positions.

## Data Availability

No datasets were generated or analysed during the current study.
